# Does the availability of snack foods in supermarkets vary internationally?

**DOI:** 10.1186/1479-5868-10-56

**Published:** 2013-05-14

**Authors:** Lukar E Thornton, Adrian J Cameron, Sarah A McNaughton, Wilma E Waterlander, Marita Sodergren, Chalida Svastisalee, Laurence Blanchard, Angela D Liese, Sarah Battersby, Mary-Ann Carter, Judy Sheeshka, Sharon I Kirkpatrick, Sandy Sherman, Gill Cowburn, Charlie Foster, David A Crawford

**Affiliations:** 1 Centre for Physical Activity and Nutrition Research, School of Exercise and Nutrition Sciences, Deakin University, Melbourne Burwood Campus, 221 Burwood Highway, Burwood, Melbourne 3125, Australia; 2 Department of Health Sciences and the EMGO Institute for Health and Care Research, Faculty of Earth and Life Sciences, VU University, Amsterdam, The Netherlands; 3 National Institute for Health Innovation, School of Population Health, The University of Auckland, Auckland, New Zealand; 4 Centre of Family Medicine, Department of Neurobiology, Care Sciences and Society, Karolinska Institutet, Huddinge, Sweden; 5 Metropolitan University College, Copenhagen, Denmark; 6 Department of Public Health, University of Copenhagen, Copenhagen, Denmark; 7 Department of Epidemiology and Biostatistics, Center for Research in Nutrition and Health Disparities, Arnold School of Public Health, University of South Carolina, Columbia, USA; 8 Department of Geography, University of South Carolina, Columbia, USA; 9 Department of Public Health, University of Otago, Wellington, New Zealand; 10 School of Biomedical and Health Sciences, Victoria University, Melbourne, Australia; 11 Division of Cancer Control and Population Sciences, US National Cancer Institute, Bethesda, USA; 12 The Food Trust, Philadelphia, USA; 13 British Heart Foundation Health Promotion Research Group, Department of Public Health, University of Oxford, Oxford, UK

**Keywords:** Snack foods, Food environment, Supermarket, International comparison

## Abstract

**Background:**

Cross-country differences in dietary behaviours and obesity rates have been previously reported. Consumption of energy-dense snack foods and soft drinks are implicated as contributing to weight gain, however little is known about how the availability of these items within supermarkets varies internationally. This study assessed variations in the display of snack foods and soft drinks within a sample of supermarkets across eight countries.

**Methods:**

Within-store audits were used to evaluate and compare the availability of potato chips (crisps), chocolate, confectionery and soft drinks. Displays measured included shelf length and the proportion of checkouts and end-of-aisle displays containing these products. Audits were conducted in a convenience sample of 170 supermarkets across eight developed nations (Australia, Canada, Denmark, Netherlands, New Zealand, Sweden, United Kingdom (UK), and United States of America (US)).

**Results:**

The mean total aisle length of snack foods (adjusted for store size) was greatest in supermarkets from the UK (56.4 m) and lowest in New Zealand (21.7 m). When assessed by individual item, the greatest aisle length devoted to chips, chocolate and confectionery was found in UK supermarkets while the greatest aisle length dedicated to soft drinks was in Australian supermarkets. Only stores from the Netherlands (41%) had less than 70% of checkouts featuring displays of snack foods or soft drinks.

**Conclusion:**

Whilst between-country variations were observed, overall results indicate high levels of snack food and soft drinks displays within supermarkets across the eight countries. Exposure to snack foods is largely unavoidable within supermarkets, increasing the likelihood of purchases and particularly those made impulsively.

## Background

There has been a continued growth in global obesity rates over recent decades although the trajectory of these increases has varied across developed nations [1,2]. The higher prevalence of obesity has put large numbers of individuals at increased risk of type 2 diabetes, cardiovascular diseases and some cancers [3-5].

Snacking behaviours have increased in parallel with obesity prevalence [6-8]. Evidence has linked the increased consumption of energy-dense snack foods with greater waist circumference in adults [9] while sugar-sweetened beverage consumption (e.g. soft drinks) is increasingly implicated in the development of obesity [10]. Although existing studies allow us to assess snack food consumption in individual countries [7,11-19], international comparison studies remain relatively rare. Those studies including data from multiple countries consistently report large differences in purchasing and/or consumption across countries [20-25].

Environmental factors including opportunities to purchase and consume food are increasingly seen as an important determinant of dietary behaviours [26]. Within food stores, consumers are faced with variations in the quantity, quality, price and marketing of food items [27-30]. Supermarkets are a major source of food for many households as they are usually highly accessible (both in terms of location and opening hours) and enjoy market domination in food/grocery retail expenditure in many developed nations (including Australia [31,32], Canada [33], Sweden [32], the US [34], and the UK [35]). As a setting for within-store food availability research, supermarkets are therefore an important priority.

Supermarket retailers have long known that the placement and marketing of products is a key determinant of purchasing decisions with marketing tactics being particularly important for sales of impulse items in comparison with staples [30,36-40]. Market research findings have demonstrated that almost two thirds of in-store food purchasing decisions are unplanned, [41] highlighting the potential for purchasing and consumption to be influenced by within-store displays and particularly those in highly visible areas (e.g. at end-of-aisles and checkouts). Our previous assessments of the supermarket snack food environment (from Melbourne, Australia) showed snack foods to be almost ubiquitous at supermarket checkouts, and extremely common in end-of-aisle displays and temporary island bins [42]. A significant amount of supermarket shelf space was also dedicated to snack foods and soft drinks as static displays in aisles [43]. The few other studies of supermarket snack foods conducted to date have been restricted to individual cities [44-48] with the exception of Farley *et al*. who conducted one study across 19 US cities [49] and another across two US cities [50]). These two US studies also concluded that snack foods were readily available within a range of retail food store types [49,50]. All previous studies have limited comparability because of the use of an assortment of measurement techniques and no international comparisons have been previously published.

In the present paper we use data gathered in a standardized manner from supermarkets in eight different countries to examine the availability of snack food (potato chips, chocolate, and confectionery) and soft drinks in the shelves, at checkouts and in end-of-aisle displays. Shelf space of fruit and vegetables were also assessed for comparative purposes. Our focus on energy-dense snack foods and soft drinks was based on the above mentioned established links between consumption of such products and health and the fact that consumption of such products is common across the eight developed countries. International comparison studies of the within-store food environment such as this are an important way of placing local findings into a global context and can provide significant insights for policy and advocacy toward healthier food environments.

## Methods

### Sampling

A total of 170 supermarkets were audited across selected cities in eight developed countries (Table 1) using a standardized audit tool (Additional file 1: Appendix A). The study involved an international collaboration with data collection in each country organised by the local research team. Many of the auditors had face-to-face contact with the lead investigators (LET & AJC) prior to the collection of data and each auditor was provided with the audit tool and a detailed instruction manual (including photographic instructions) (Additional file 1: Appendix A). A more expansive audit tool was used for the data collection in Australia, Canada (Montreal only), Denmark, and the Netherlands but some measures were removed (e.g. variety, price, island bin displays) for the remaining countries to reduce auditor burden and to keep only those features that were readily comparable between countries. Auditors contacted the lead investigators with any questions that arose during data collection.

**Table 1:** Sample characteristics of the 170 included supermarkets from 8 countries

**Country**	**City/region**	**No.**	**Supermarket chains audited (n)**	**Dates of audit**
Australia	Melbourne	35	Coles (16), Woolworths (19)	Sept 2010 – Nov 2010 & Feb 2011
Canada	Montreal (18); Toronto (10)	28	Food Basics (1), Highland Farms (1), IGA (2), IGA Extra (2), Les Marchés Traditions (1), Loblaws Superstore (1), Longo’s (1), Marché Ami (1), Métro (8), Métro Plus (2), Michael-Angelo’s (1), No Frills (1), Price Chopper (1), Provigo (4), Super C (1)	Nov 2011, Jan 2012 & June 2012
Denmark	Copenhagen and surrounding areas	18	Fakta (4), Fotex (1), Irma (3), Lidl (1), Netto (3), Rema 1000 (1), Spar (1), Superbest (3)	Aug- Sept 2011
Netherlands	Amsterdam	20	Albert Heijn (20)	May – June 2011
New Zealand	Wellington	10	Countdown (3), New World (5), Pak n Save (2)	July 2012
Sweden	Stockholm	19	Coop (8), ICA (11)	July 2011 & June 2012
United Kingdom	Oxford	8	Co-op (1), Marks and Spencer (1), Sainsbury (3), Tesco (2), Waitrose (1)	Feb 2012
United States of America	Columbia (14); Philadelphia (9); Bethesda/Washington DC (9)	32	Acme (1), Bi-Lo (3), Bottom Dollar (1), Fresh Grocer (2), Fresh Market (1), Food Lion (5), Giant (4), Harris Teeter (2), Kroger (1), Piggly Wiggly (3), Safeway (4), Sams (1), Save a Lot (1), Shop n Bag (1), Superfresh (1), Trader Joes (1)	June-July 2012

The data collection for Australia took place between September 2010 and February 2011 whilst the audits for the other countries were conducted between May 2011 and July 2012. In Australia [43], Denmark, the Netherlands and Canada (Montreal only) supermarkets were sampled from neighbourhoods within the least and most socioeconomically disadvantaged areas. While auditors were instructed to sample from a representative range of areas, no explicit sampling criteria were followed and auditors sampled from their cities of residence or other region convenient to them. Therefore, the actual areas sampled should be considered a convenience sample.

The local supermarket retailers from each region were included in the audits. In some countries this meant only a limited number of chains were sampled whereas elsewhere multiple chains were sampled as this reflected the increased diversity with the local supermarket industry.

The project proposal for data collection in Melbourne was assessed by a Human Research Ethics Advisor from the Office of Research Integrity at Deakin University who advised that ethics committee approval for the study was unnecessary because data collection did not involve personal disclosure. Ethical assessment was the responsibility of each collaborator where it was required by their host institutions. In each instance ethics approval was not required as this research did not involve human participants. Auditors gained consent from store managers prior to taking any measurements within a store.

### Data collection

#### Items included

A universal definition of “snack food” does not exist [51,52], and for the purpose of this research we restricted our definition to food and beverage types that are often consumed outside of the three main meals and would be considered high in energy, high in sodium, and/or low in micronutrients. These items included potato chips (crisps) (includes corn chips but not savoury crackers or pretzels), chocolate (either as chocolate bars, blocks, boxes or bags), confectionery (candy) (excluding mints and chewing gum) and soft drinks (soda) (both diet and regular). Each of these product types were easily identified in distinct sections in most supermarkets, readily available in the eight included countries, and form part of a typical Western-style eating pattern. Both diet and regular soft drinks were included because they are often interspersed within the same shelves (i.e. not stocked in separate sections) preventing us from taking separate aisle length measures for these two products. Fruit and vegetable availability was also measured (fresh products only, not tinned, dried or frozen) to allow an assessment of the ratio of snack foods to fruits and vegetables within stores.

#### Shelf space

The total aisle length (in metres) dedicated to each of the four snack food and beverage groups (i.e. potato chips, chocolate, confectionery and soft drinks) was measured using a measuring wheel or measuring tape. This involved measuring from the point in the aisle where the snack food category began to where the display of that category ended. In the stores from the Bethesda/Washington DC area, a measuring device was not available and instead the length was determined by calibrated length of paces. If an item (e.g. confectionery) was displayed in multiple aisles, the total length across the multiple aisles was summed. For fruits and vegetables, the length of shelf space (refrigerated and un-refrigerated) as well as the circumference of island displays dedicated to fresh fruits and vegetables were measured and summed.

#### Checkouts and end-of-aisle displays

Auditors assessed whether snack foods were available at each checkout and at end-of-aisle displays at both the front and the back of an aisle. Using a checklist, the presence of each of the following items was recorded: 1) potato chips; 2) chocolate; 3) confectionery; 4) soft drink – regular; 5) soft drink –diet. Multiple item types could be recorded for each checkout or end-of-aisle display.

#### Store size

Total store size was calculated as total length of all aisles in the supermarket measured using a measuring wheel, measuring tape, or calibrated paces.

### Analysis

The estimated marginal mean of the total aisle length of snack food items and the aisle length of individual snack food items was calculated adjusting for total store size across all countries. The ratio of aisle length dedicated to snack foods compared to fruits and vegetables was calculated by dividing the total aisle length of snack foods by the total length of displays of fruits and vegetables. Shelf length of each product type and the ratio of snack food to fruits and vegetables for each country were ranked from the most (1) to the least (8). The proportion of total snack food aisle length dedicated to each of the snack food and soft drink items was calculated for each store to produce a within-country mean. This measure allowed us to assess the relative amount of each item within countries. For checkouts and end-of-aisle displays, results are reported as a percentage (and 95% confidence interval) of the total number of checkouts or end-of-aisle displays that displayed any snack food item.

## Results

Included data originated from Oceania, North America and Europe. In Oceania, in addition to a sample of 35 supermarkets from Melbourne, Australia on which we have previously reported [42,43], data was also obtained from 10 supermarkets in Wellington (New Zealand). A sample of 60 North American supermarkets were obtained from two cities in Canada (Montreal (n = 18) and Toronto (n = 10)) and three cities in the United States (US) (Columbia, SC (n = 14); Philadelphia, PA (n = 9); and Bethesda, MD/Washington DC (n = 9)). In Europe, audits were conducted in Copenhagen and surrounding urban areas (Denmark, n = 18), Amsterdam (the Netherlands, n = 20), Stockholm (Sweden, n = 19) and Oxford (United Kingdom (UK), n = 8) (Table 1).

### Shelf space

Aisle length dedicated to each snack food type is presented in Table 2 in addition to the country ranking for each item relative to other countries. Large variations for each snack food type as well as the total aisle length of snack food were observed between countries. Adjusted for total store size, supermarkets in the UK sample had the greatest aisle length dedicated to chips, chocolate and confectionery as well as the greatest total snack food aisle length (56.4 m; 95% CI 47.6 – 65.2 m). Aisle length of soft drinks was greatest in the Australian sample (18.4 m; 95% CI 16.6 – 20.3 m). Stores in the New Zealand sample had the least aisle length dedicated to snack foods (21.7 m; 95% CI 13.8 – 29.7). Raw aisle length (unadjusted for store size) values for each country are found in Additional file 2: Appendix B. Stores in the Danish and the UK samples had the highest ratio of snack foods to fruit and vegetables with a ratio of 1.46 and 1.31 respectively (Table 2). The Canadian supermarkets had the lowest ratio of snack food aisle length to fruits and vegetables aisle length.

**Table 2:** Shelf space (aisle length in metres) dedicated to energy-dense snack foods and drinks (adjusted for total store size) and ratio of snack food to fruits and vegetables and between-country rankings for each item

**Within-country mean length (and 95% CI of aisle displays (metres))**							
**Country**	**Chips**^**1**^	**Chocolate**^**1**^	**Confectionery**^1^	**Soft drink**^**1**^	**Total snack foods**^**1**^	**Ratio (snack/F&V)**	**Total store size**
Australia^2^	12.5 (10.8-14.1)	10.0 (8.6-11.4)	5.28 (3.8-6.8)	18.4 (16.6-20.3)	45.8 (41.4-50.1)	0.58 (0.4-0.7)	262.2 (238.6-285.7)
(rank)	*5*	*2*	*4*	*1*	*2*	*4*	*3*
Canada	13.9 (12.1-15.7)	4.91 (3.4-6.4)	3.51 (1.9-5.1)	12.6 (10.6-14.6)	34.9 (30.2-39.6)	0.33 (0.1-0.5)	188.3 (158.8-217.8)
(rank)	*3*	*6*	*7*	*4*	*5*	*8*	*5*
Denmark	11.3 (9.1-13.6)	7.92 (6.0-9.9)	7.27 (5.2-9.3)	12.2 (9.7-14.7)	38.7 (32.7-44.7)	1.46 (1.3-1.6)	128.3 (94.7-161.9)
(rank)	*6*	*4*	*3*	*5*	*4*	*1*	*6*
Netherlands	10.6 (8.3-12.9)	5.31 (3.38-7.2)	3.85 (1.8-5.9)	11.1 (8.61-13.6)	30.5 (24.6-36.4)	0.44 (0.2-0.6)	76.9 (59.3-94.6)
(rank)	*7*	*5*	*6*	*6*	*7*	*7*	*8*
New Zealand	7.17 (4.3-10.2)	2.32 (-0.2-4.9)	3.98 (1.3-6.7)	8.20 (4.8-11.5)	21.7 (13.8-29.7)	0.50 (0.4-0.7)	279.9 (212.0-347.7)
(rank)	*8*	*8*	*5*	*8*	*8*	*6*	*2*
Sweden	14.5 (12.2-16.8)	9.23 (7.3-11.1)	9.33 (7.3-11.3)	10.8 (8.3-13.3)	43.8 (37.8-49.8)	0.72 (0.5-0.9)	93.4 (59.9-126.8)
(rank)	*2*	*3*	*2*	*7*	*3*	*3*	*7*
United Kingdom	15.2 (11.8-18.5)	15.6 (12.7-18.4)	11.6 (8.6-14.6)	13.9 (10.2-17.7)	56.4 (47.6-65.2)	1.31 (1.0-1.6)	232.0 (39.8-424.2)
(rank)	*1*	*1*	*1*	*3*	*1*	*2*	*4*
United States of America	12.9 (11.1-14.7)	2.87 (1.3-4.4)	2.81 (1.2-4.4)	14.0 (12.0-16.0)	32.7 (28.0-37.5)	0.56 (0.4-0.7)	307.8 (212.0-347.7)
(rank)	*4*	*7*	*8*	*2*	*6*	*5*	*1*

^1 ^adjusted for store size.

^2 ^shelf space reported in our previous analysis of Australian supermarkets [43] will differ to the results presented here as the current analysis accounts for differences in store size between countries.

**Figure 1:**
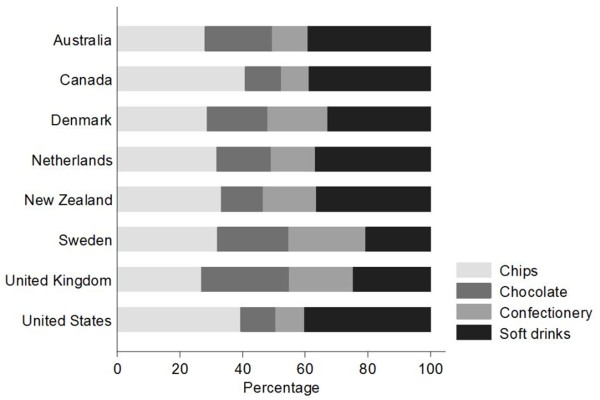
Proportion of total snack food aisle length dedicated to each snack food item.

The proportion of the total snack food aisle length dedicated to each type of snack food is presented in Figure 1. Supermarkets in the North American countries (Canada and the US) had a greater proportion of their snack food aisle length dedicated to chips. Compared to other countries, the UK sample had a higher proportion of total snack food shelf length allocated to chocolate while the greatest proportion of shelf length allocated to confectionery was found within the Swedish sample. Soft drinks were less prominent as a proportion of total snack food aisle length in the Swedish and UK supermarkets.

### Checkouts and end-of-aisle displays

**Figure 2:**
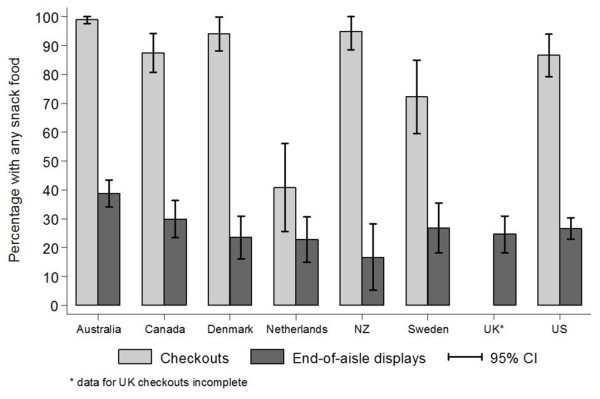
Mean within-store percentage (and 95% CI) of checkouts and end-of-aisle displays with any snack food present.

The only sample of supermarkets in which less than half of all checkout displays featured any of the snack foods or soft drinks assessed were those in the Netherlands (41%) (Figure 2). In every other country, our samples of supermarkets had more than 70% of checkouts featuring snack foods or soft drinks, with the mean percentage being highest in Australia (99%). The total number of checkouts in the UK sample was not recorded and therefore we could not calculate the proportion of checkouts with snack foods within that country. The percentage of checkout displays in each country featuring each of the individual product types is presented in Additional file 2: Appendix C. Of particular interest in this appendix is the diversity of snack foods available at the checkouts with the US sample compared to countries such as Australia where checkouts largely display chocolate or soft drinks.

Australian supermarkets also exhibited the highest percentage (39%) of end-of-aisle displays featuring snack foods or soft drinks, with the proportion from the samples in other countries ranging from 16% (New Zealand) to 30% (Canada) (Figure 2). Additional file 2: Appendix D contains the percentage of end-of-aisle displays in each country featuring each of the individual product types.

## Discussion

This study investigated the exposure to energy-dense snack foods (chips, chocolates and confectionery) and soft drinks in a sample of supermarkets across selected cities in eight developed countries. The shelf length of snack foods and the presence of these foods at checkouts and in end-of-aisle displays were assessed with noticeable variations detected between countries. UK supermarkets had the greatest aisle length devoted to chips, chocolate and confectionery, while soft drink aisle length was greatest in Australia. The proportion of both checkouts and end-of-aisle displays containing snack food was also highest in the Australian supermarkets sampled. In every country other than the Netherlands, snack foods were present at over 70% of checkouts.

From the results of this cross-sectional study, we are not able to discern whether variation is a result of differences in demand between countries, or whether supply is driving demand. It is likely that both are contributing. Demand may be driven strongly by cultural norms and traditional diet preferences. Influences on the supply side of the equation may include specific commercial arrangements between retailers and the food industry/suppliers, climatic differences between countries that influence the availability and price of supermarket items, agricultural policies such as the subsidisation of high-fructose corn-syrup production (for use as a sweetener in soft drinks) by the US government [53], import tariffs and trade agreements. The position of supermarkets in the larger food shopping environment may also explain some of the variation observed here. For example, it is likely the role of supermarkets in supplying fresh fruit and vegetables to consumers varies between nations based on the prominence and use of other retailers such as greengrocers or markets. Such variations may have contributed to the differences in the ratio of snack foods to fruits and vegetables that we observed.

Some of the findings reported here can be compared with the results of previous studies from individual countries. A 2006 study from Melbourne, Australia reported that 99% of checkouts displayed snack foods or soft drinks [45] which is consistent with our own findings from that city. Prior US research [46,50] using a more inclusive definition of snack foods (included such items as nuts, cookies, doughnuts) found greater shelf lengths dedicated to snack food items than we report here. Because of the differences in definitions, the results from this study and our own study are not directly comparable.

Whilst the broader dietary and health implications of supermarkets have been discussed elsewhere [29,30], the link between snack food availability and both purchasing behaviour and health indicators warrants consideration. Although research in this area is in its infancy, a couple of studies have been published. One prior Australian study did not find any link between snack food shelf length and purchasing [54]; however that study of only nine supermarkets was underpowered to detect a significant effect. In the US, a positive correlation between snack food shelf space and BMI was observed [55] however the effect size was small. Many policy and program interventions are already aiming to change food environments even though robust and consistent evidence is not yet available and a consensus on how change should best be achieved has not yet been reached [27]. Additional studies linking within-store environments with purchasing habits, diets and obesity are therefore sorely required to support the obvious desire to improve our food environment.

While ecological data of the type reported from this study cannot infer causality, links between availability and national consumption patterns are of interest. In Dutch supermarkets, greater shelf space was allocated to soft drinks in comparison with the other snack foods assessed. This result correlates with the findings from the ENERGY study (which examines health in children across seven European countries) in which extremely high soft drink consumption amongst Dutch children was reported [20]. Furthermore, soft drink consumption in the Netherlands has increased by 74% between 1980 and 2009 [56]. In the North American supermarkets audited, shelf space devoted to soft drinks and potato chips was greater than for confectionery and chocolate in comparison with the other countries assessed. Within the US, soft drinks (soda) were reported to be the top dietary source of added sugars [57,58] whilst potato chips were the top dietary source of oils [58]. Amongst U.S. children both soft drinks and potato chips make substantial contributions to overall energy intake [57]. The Swedish population has traditionally had a preference for sugar confectionery [59]. Of interest however, are changes between 1980 and 2010 in the consumption of different snack foods and drinks. In that period, chocolate and confectionery (combined) increased by 53% per capita in Sweden whilst the consumption of soft drinks (including flavoured carbonated water) over this period increased three-fold and consumption of potato chips increased four-fold [60]. These changes in consumption are reflected in the current snack food profile in Swedish supermarkets in which shelf space of confectionery and chocolate is no higher than for chips and soft drinks (noting that our measure does not include carbonated water). Although it is of interest to examine correlations between availability and consumption patterns, the lack of comparable national dietary indicators limits our ability to explore this in more detail.

Whilst limiting snack food exposure in other settings such as schools and workplaces has been a focus of some public health campaigns [61,62], the supermarket environment is increasingly recognised as a potential intervention point [29]. In addition to facilitating comparisons between countries, the results of this study also allow the assessment of the local food environment in each country. Such national food environment data is necessary to support and justify campaigns such as those calling for the removal of confectionery items from supermarket checkouts [61,63,64]. Efforts to improve diet and reduce obesity and other chronic diseases will be more successful when supported by strategies such as these that aim to create healthier environments. In the Netherlands, the Albert Heijn supermarket chain has the largest market share (34%) [65] and remains a profitable supermarket retailer despite not having high levels of snack food displays (relative to other supermarkets in this study). In reaction to a report by the Dutch Consumers’ Federation [66] on which supermarkets make the healthy choice the easy choice, Albert Heijn announced an initiative to remove all snack foods from checkouts. This action is reflected in the low proportion of Dutch checkouts with snack food displays observed in this study. That example suggests that such initiatives may involve a relatively low cost for supermarkets, can allow them to promote their brand as a healthy choice, and may be important in changing cultural norms around snacking behaviours. Other forms of advocacy to encourage healthier supermarkets are required [29] and these may include policy-level approaches initiated by governments.

The major strength of this study is the within-store assessment of energy-dense snack foods and soft drinks conducted in a sample of supermarkets across cities in multiple countries using a standardized measurement tool. Multiple aspects of the within-store food environment were captured, including static displays of shelf space and dynamic displays at ends-of-aisles and checkouts. Previously, within-store assessments of supermarket snack foods have been rare, limited in their ability to compare findings between countries and did not include the multiple aspects of the supermarket snack food environment assessed here.

We acknowledge that our definition of snack food was limited to four food categories. Other types of energy-dense snack foods not captured here are also available in supermarkets and in certain contexts these products may also be important snack foods (e.g. cookies and ice-cream in North America). While no universal definition of snack foods exists [51,52], the definition used in this study was appropriate for a cross-country comparison as each of the snack foods examined were commonly available in all countries. The sampling strategy should also be considered when interpreting the findings. In Melbourne, Amsterdam and Montreal, data collection was undertaken according to area level disadvantage. Auditors in other countries sampled from a representative range of urban areas with no pre-specified stratification according to area-level disadvantage. All collaborators were instructed to sample from the major supermarket retailers in their setting. Although this meant that a greater variety of chains were sampled in some countries than others, we would expect that the diversity of store types in a country is a valid reflection of the choices available to consumers. The fourth limitation relates to the relatively small number of supermarkets examined in two of the countries (New Zealand and the United Kingdom). The findings from these two countries in particular should be treated with some caution as they may not accurately represent snack food availability in those settings. Despite adjusting for store size in our analysis, we did not have any indicator of the presence of non-food items present and it is possible that in some larger stores, a greater proportion of the store was allocated to such products. We did, however include an assessment of the ratio of snack foods to fruits and vegetables which is an effective indicator of the priority given to snack food relative to other items. Finally, other factors that may be important determinants of snack food purchasing (e.g. price, in-store promotions, variety, island bin displays) were not included in this study because of difficulties in comparing such features between countries.

## Conclusion

Globally, supermarkets play an increasingly important role in shaping dietary behaviours. This study has highlighted the ubiquitous presence of energy-dense snack food items in the supermarkets of eight developed countries. Although differences were observed between countries, snack food was extremely common in the aisles of supermarkets in all countries. The prominence of snack foods in displays at checkouts and the ends-of-aisles may be an important determinant of snack purchases as such displays are largely unavoidable. The relatively low prominence of snack food in supermarkets in both the Netherlands (checkouts in particular) and New Zealand suggest that lessons about the reduction of such displays may be learnt from these countries.

## Competing interests

The authors declare that they have no competing interests.

## Authors’ contributions

LET and AJC designed the audit tool with the assistance of SAM. LET and AJC oversaw the data collection within Melbourne and analysed the data for the entire sample. All other listed authors either coordinated or conducted the data collection within their country. LET and AJC wrote the first draft of the paper with the assistance of SAM and DAC. All authors assisted in drafting the manuscript with each author providing context-specific contributions. LET had primary responsibility for the final content. All authors read and approved the final manuscript.

## Supplementary Material

Additional file 1 **Appendix A. **Supermarket snack food audit tool. *Please note the audit tool is freely available for other researchers to use (instruction manual also freely available on request to the corresponding author). Please acknowledge the audit tool developers when reporting findings based on the use of this audit tool. Click here for file

Additional file 2 **Appendix B. **Raw mean (unadjusted) shelf length of snack food items, soft drinks and fruits and vegetables (fruit and vegetable shelf length adjusted for total store size also presented). **Appendix C. **Percentage of checkouts in 170 supermarkets from 8 countries displaying individual snack food items and soft drinks. **Appendix D. **Percentage of end-of-aisles (front of aisle, back of aisle and total) displaying individual snack food items and soft drinks. Click here for file
