# Supplementary material for: Does the availability of snack foods in supermarkets vary internationally?
Source: Int J Behav Nutr Phys Act. 2013 May 14;10:56. doi: 10.1186/1479-5868-10-56 (PMC3660266; doi:10.1186/1479-5868-10-56)
Supplement: Additional file 1 — Appendix A. Supermarket snack food audit tool. *Please note the audit tool is freely available for other researchers to use (instruction manual also freely available on request to the corresponding author). Please acknowledge the audit tool developers when reporting findings based on the use of this audit tool. [file 1479-5868-10-56-S1.pdf]

Supermarket Snack Food Audit

1a) Auditor initials ______

1b) Date of audit ______

1c) Time of audit start ______

1d) Time of audit finish ______

1e) Supermarket chain: ____

1f) Consent gained ☐

1g) Supermarket address ____

2) Fresh fruit and Vegetables

Shelf space audit:

2a) Length of fridge/chilled shelf containing vegetables (in metres)________________

2b)

**Island bins**	
Circumference	Number of bins

3) Supermarket layout audit – length of aisle and end of aisle displays

Please record the product at the front (near register) and back of each aisle, plus aisle length. (multiple items can be recorded)

**Aisle Number**<br>If aisle is split, renumber as 1a, 1b, 2a, 2b, etc.	**Aisle length (m)**<br>**a)** same as previous	**Product: Front**<br>**a)** reg soft drink<br>**b)** diet soft drink<br>**c)** chips<br>**d)** chocolate<br>**e)** confectionery<br>**f)** fruit/veg<br>**g)** non-snack item<br>**h)** no end-of-aisle display	**Product: Back**<br>**a)** reg soft drink<br>**b)** diet soft drink<br>**c)** chips<br>**d)** chocolate<br>**e)** confectionery<br>**f)** fruit/veg<br>**g)** non-snack item<br>**h)** no end-of-aisle display
1			
2			
3			
4			
5			
6			
7			
8			
9			
10			
11			

If store is non-standard in terms of shape/ setup draw a picture below to help indicate how you measured store size (e.g. total store length x width) and which side of the aisle you considered as the front and the back of store (if the aisles run parallel with the checkouts).

Aisle Number	**Aisle length (m)**<br>**a)** same as previous<br>**b)** last aisle	**Product: Front**<br>**a)** reg soft drink<br>**b)** diet soft drink<br>**c)** chips<br>**d)** chocolate<br>**e)** confectionery<br>**f)** fruit/veg<br>**g)** non-snack item	**Product: Back**<br>**a)** reg soft drink<br>**b)** diet soft drink<br>**c)** chips<br>**d)** chocolate<br>**e)** confectionery<br>**f)** fruit/veg<br>**g)** non-snack item
12			
13			
14			
15			
16			
17			
18			
19			
20			
21			
22			
23			
24			

4) Shelf space

4a) Shelf space audit – soft drinks & mineral/soda water

Note: The following are **<u>not</u>** included in the definition of soft drinks or mineral/soda water for this survey: 1) sports drinks, 2) energy drinks, 3) vitamin water, 4) juices, 5) ciders, 6) cordial, 7) bottled still water

Total aisle length of soft drinks (m): _________

Total aisle length of natural mineral and soda water drinks (m): _________

4b) Shelf space audit – chips

Total aisle length of chips (m) (including multipacks): _________

Aisle length of multipack chips only (m): _________

4c) Shelf space audit – chocolate

Total aisle length of chocolate: _________

Total aisle length of confectionery: _________

5) Cash Register audit

Record the variety of products at each cash register display (multiple items can be recorded)

**Cash register display**	**Product**<br>**a)** reg soft drink<br>**b)** diet soft drink<br>**c)** chips<br>**d)** chocolate<br>**e)** confectionery<br>**f)** kids toys<br>**g)** no snack food
1	
2	
3	
4	
5	
6	
7	
8	
9	
10	

**Cash register display**	**Product**<br>**a)** reg soft drink<br>**b)** diet soft drink<br>**c)** chips<br>**d)** chocolate<br>**e)** confectionery<br>**f)** kids toys<br>**g)** no snack food
11	
12	
13	
14	
15	
16	
17	
18	
19	
20	

**Cash register display**	**Product**<br>**a)** reg soft drink<br>**b)** diet soft drink<br>**c)** chips<br>**d)** chocolate<br>**e)** confectionery<br>**f)** kids toys<br>**g)** no snack food
21	
22	
23	
24	
25	
26	
27	
28	
29	
30	
