# Supplementary material for: Does the availability of snack foods in supermarkets vary internationally?
Source: Int J Behav Nutr Phys Act. 2013 May 14;10:56. doi: 10.1186/1479-5868-10-56 (PMC3660266; doi:10.1186/1479-5868-10-56)
Supplement: Additional file 2 — Appendix B. Raw mean (unadjusted) shelf length of snack food items, soft drinks and fruits and vegetables (fruit and vegetable shelf length adjusted for total store size also presented). Appendix C. Percentage of checkouts in 170 supermarkets from 8 countries displaying individual snack food items and soft drinks. Appendix D. Percentage of end-of-aisles (front of aisle, back of aisle and total) displaying individual snack food items and soft drinks. [file 1479-5868-10-56-S2.docx]

**Appendix B**

|  | **Unadjusted mean length (and 95% CI of aisle displays (metres))** | | | | | | | | | | | | **Fruit and Vegetables (adjusted for store size)** | | **Total store size** | |  |
| --- | --- | --- | --- | --- | --- | --- | --- | --- | --- | --- | --- | --- | --- | --- | --- | --- | --- |
| **Country** | **Chips** | | **Chocolate** | | **Confectionery** | | **Soft drink** | | **Total snack foods** | | **Fruit and Vegetables** | |  |  |  |  |  |
| Australia | 14.5 (13.1-15.9) | | 11.2 (10.2-12.1) | | 5.9 (5.4-6.5) | | 20.4 (18.2-22.5) | | 52.0 (47.8-56.1) | | 100.2 (88.4-111.9) | | 89.6 (77.8-101.3) | | 262.2 (238.6-285.7) | |  |
| Canada | 13.3 (10.6-16) | | 4.6 (2-7.2) | | 3.4 (2-4.7) | | 12 (9.7-14.4) | | 33.3 (26-40.6) | | 113.7 (90.1-137.4) | | 116.4 (103.6-129.3) | | 188.3 (158.8-217.8) | |  |
| Denmark | 8.4 (6.4-10.4) | | 6.4 (3.9-9) | | 6.5 (3.9-9) | | 9.3 (7.8-10.9) | | 30.7 (25.2-36.1) | | 27.9 (18.9-37) | | 41.5 (25.1-57.8) | | 128.3 (94.7-161.9) | |  |
| Netherlands | 5.7 (4.3-7.1) | | 2.8 (2.2-3.3) | | 2.4 (1.8-3) | | 6.1 (4.8-7.4) | | 17 (13.5-20.5) | | 35.1 (27.8-42.4) | | 57.9 (41.7-74.1) | | 76.9 (59.3-94.6) | |  |
| New Zealand | 10.1 (7.4-12.9) | | 3.9 (3.1-4.6) | | 4.9 (3.7-6) | | 11.1 (8-14.2) | | 30 (23.2-36.7) | | 69.8 (48.6-91.1) | | 56.1 (34.4-77.8) | | 279.9 (212.0-347.7) | |  |
| Sweden | 10.2 (6.9-13.5) | | 7.1 (5.1-9.1) | | 8.1 (4.5-11.8) | | 6.7 (4.2-9.1) | | 32.1 (21.6-42.5) | | 45.3 (31.4-59.1) | | 65.1 (48.8-81.4) | | 93.4 (59.9-126.8) | |  |
| United Kingdom | 16.3 (6.1-26.5) | | 16.2 (9.8-22.6) | | 12 (4.5-19.4) | | 15.1 (5.7-24.5) | | 59.6 (35-84.1) | | 56.7 (28.2-85.2) | | 51.5 (27.5-75.5) | | 232.0 (39.8-424.2) | |  |
| United States of America | 17 (14.1-19.9) | | 5 (3.5-6.5) | | 4 (3.1-4.9) | | 18 (14.5-21.5) | | 44 (36.4-51.6) | | 97.2 (81-113.4) | | 78.4 (65.5-91.2) | | 307.8 (212.0-347.7) | |  |
|  | |  | |  | |  | |  | |  | |  | |  | |  | |

**Appendix C**

|  | **Percentage of checkout displays containing each product (95% CI)** | | | | |
| --- | --- | --- | --- | --- | --- |
| **Country** | **Chips** | **Chocolate** | **Confectionery** | **Soft drink - regular** | **Soft drink - diet** |
| Australia | 1.9 (0-5.7) | 66.9 (61.5-72.2) | 3.2 (0.8-5.6) | 40.7 (35.5-46.0) | 40.3 (34.8-45.7) |
| Canada | 11.6 (6.4-16.9) | 69.6 (61.1-78.0) | 37.7 (24.8-50.7) | 23.1 (15.3-30.8) | 23.3 (15.6-31.0) |
| Denmark | 0 | 50.1 (29.5-70.7) | 89.1 (82.3-95.9) | 4.2 (0-8.9) | 1.9 (0-5.5) |
| Netherlands | 2.0 (0-5.9) | 36.5 (22.3-50.7) | 20.5 (7.9-33.2) | 4.3 (0-9.1) | 2.3 (0-5.3) |
| New Zealand | 0 | 83.0 (71.6-94.4) | 87.0 (79.2-94.8) | 28.2 (13.6-42.9) | 25.5 (14.6-36.3) |
| Sweden | 12.5 (0-25.3) | 41.6 (27.8-55.5) | 62.3 (50-74.7) | 31.4 (17.9-44.9) | 17.3 (7.4-27.3) |
| United Kingdom^1^ | n/a | n/a | n/a | n/a | n/a |
| United States of America | 27.5 (18.2-36.9) | 76.4 (69.1-83.7) | 49.3 (34.9-63.7) | 52.5 (41.9-63.0) | 49.2 (38.5-59.9) |
| ^1^ The total number of checkouts in the UK sample was not recorded and therefore we could not calculate the proportion of checkouts with snack foods within that country | | | | | |

**Appendix D**

|  | | | **Percentage of End-of-Aisle displays containing each product (95% CI)** | | | | | | | | | | | | |
| --- | --- | --- | --- | --- | --- | --- | --- | --- | --- | --- | --- | --- | --- | --- | --- |
| **Country** | | **Chips** | | | | | | **Chocolate** | | | | | **Confectionery** | | |
|  | | Front | | Back | | Total | | Front | Back | | Total | | Front | Back | Total |
| Australia | | 5.8 (3.6-7.9) | | 4.7 (2.6-6.8) | | 5.2 (3.6-6.9) | | 12.8 (8.4-17.2) | 28.2 (20.1-36.3) | | 20.5 (15.8-25.2) | | 2.8 (1.2-4.4) | 8.4 (4.8-12.0) | 5.6 (3.6-7.6) |
| Canada | | 12.9 (9.8-16.1) | | 15.1 (10.9-19.2) | | 14.0 (12.0-16.0) | | 8.9 (4.2-13.5) | 5.9 (2.5-9.4) | | 7.4 (4.2-10.6) | | 9.1 (4.7-13.4) | 7.2 (3.0-11.4) | 8.1 (4.5-11.8) |
| Denmark | | 1.1 (0-2.7) | | 5.5 (2.5-8.6) | | 3.3 (1.8-4.8) | | 12.5 (6.2-18.8) | 4.9 (1.8-8.0) | | 8.7 (5.4-12.1) | | 8.3 (2.0-14.6) | 5.8 (1.8-9.7) | 7.0 (3.9-10.1) |
| Netherlands | | 3.2 (0-6.5) | | 7.0 (1.9-12.1) | | 5.1 (2.1-8.1) | | 4.1 (0.9-7.3) | 1.6 (0-3.4) | | 2.9 (1.1-4.6) | | 1.3 (0-3.7) | 0 | 0.6 (0-1.9) |
| New Zealand | | 10.4 (0-21.0) | | 6.3 (0.3-12.3) | | 8.4 (0.3-16.4) | | 9.1 (2.8-15.5) | 1.4 (0-3.4) | | 5.3 (2.3-8.2) | | 2.9 (-2.8-8.5) | 0 | 1.4 (0-4.2) |
| Sweden | | 7.2 (2.3-12.1) | | 4.4 (1.1-7.7) | | 5.8 (3.2-8.4) | | 14.6 (9.7-19.6) | 7.2 (1.8-12.6) | | 10.9 (6.7-15.1) | | 9.0 (2.3-15.7) | 4.2 (1.6-6.7) | 6.6 (3.1-10.1) |
| United Kingdom | | 8.2 (2.1-14.3) | | 2.7 (0-5.6) | | 5.5 (1.9-9.1) | | 16.5 (5.1-28.0) | 14.7 (2.7-26.7) | | 15.6 (4.3-26.9) | | 14.9 (4.1-25.7) | 16.2 (0-33.5) | 15.6 (7.1-24.1) |
| United States of America | | 12.1 (8-16.1) | | 15.7 (11.7-19.7) | | 13.9 (11.0-16.8) | | 1.0 (0-2.2) | 1.4 (0.4-2.4) | | 1.2 (0.4-2.1) | | 0.3 (0-1.0) | 0.8 (0-1.7) | 0.6 (-0.2-1.3) |
| **Country** | **Soft drink - regular** | | | | | | **Soft drink - diet** | | | | |  |  |  |  |
|  | Front | | | Back | | Total | Front | | Back | | Total |  |  |  |  |
| Australia | 23.0 (20.5-25.4) | | | 7.3 (4.3-10.4) | | 15.1 (13.0-17.3) | 18.9 (16.5-21.4) | | 3.7 (1.5-6.0) | | 11.3 (9.5-13.1) |  |  |  |  |
| Canada | 4.1 (1.5-6.7) | | | 8.3 (3.6-12.9) | | 6.2 (3.4-8.9) | 3.4 (1.0-5.8) | | 7.5 (2.8-12.2) | | 5.4 (2.7-8.2) |  |  |  |  |
| Denmark | 2.1 (0-4.2) | | | 4.8 (0.4-9.3) | | 3.4 (1.1-5.8) | 0.6 (0-1.8) | | 0 | | 0.3 (0-0.9) |  |  |  |  |
| Netherlands | 13.5 (7.3-19.7) | | | 8.1 (3.5-12.8) | | 10.8 (7.6-14.0) | 10.3 (5.5-15.1) | | 8.1 (3.5-12.8) | | 9.2 (6.5-12) |  |  |  |  |
| New Zealand | 8.8 (0-19.8) | | | 5.7 (0-11.9) | | 7.3 (0-15.4) | 6.0 (0.2-11.7) | | 2.9 (0-6.5) | | 4.4 (1.4-7.4) |  |  |  |  |
| Sweden | 6.8 (2.1-11.5) | | | 9.4 (0-19.9) | | 8.1 (1.3-14.9) | 4.2 (0.4-8.0) | | 0.4 (0-1.2) | | 2.3 (0.3-4.3) |  |  |  |  |
| United Kingdom | 7.6 (1.1-14.1) | | | 6.2 (0.5-11.9) | | 6.9 (2.9-10.9) | 2.9 (0.5-5.2) | | 1.0 (0-2.3) | | 1.9 (0.5-3.4) |  |  |  |  |
| United States of America | 9.7 (6.5-13.0) | | | 15.0 (11.1-18.9) | | 12.4 (9.2-15.6) | 9.4 (6.3-12.5) | | 11.8 (8.5-15.1) | | 10.6 (7.9-13.2) |  |  |  |  |
